# Rotator cuff injury and myocardial infarction: Findings of NHANES 2011 to 2018 and Mendelian randomization study

**DOI:** 10.1097/MD.0000000000045840

**Published:** 2025-11-07

**Authors:** Zhi Wang, Jianjun Dang, Liang Xu, Xin Yang, Chengming Jia

**Affiliations:** a Shaanxi University of Chinese Medicine, Xianyang, China; b Department of Orthopedics, Shaanxi Provincial Hospital of Chinese Medicine, Xi’an, China.

**Keywords:** Mendelian randomization, myocardial infarction, NHANES, rotator cuff injury

## Abstract

Rotator cuff injury (RCI) and myocardial infarction (MI) are prevalent conditions in clinical practice. Nevertheless, the causal relationship between the 2 remains poorly understood. This study aimed to explore the complex association between RCI and MI by integrating cross-sectional analysis with Mendelian randomization (MR) approaches. The cross-sectional study utilized data from the National Health and Nutrition Examination Survey spanning 2011 to 2018, which involved in 567,36 participants. It assessed the correlation between RCI and MI using Chi-square tests, *T* tests, and a multiple logistic regression model. Additionally, we conducted MR analysis to investigate the causal effects of RCI on MI. We employed inverse variance weighted (IVW), sensitivity analysis, heterogeneity testing, and other methods for MR. The RCI data was sourced from the FinnGen (n = 390,666), while the aggregated data on MI was obtained from genome-wide association studies statistics (n = 638,000). In this cross-sectional analysis, after adjusting for age, gender, race, smoking status, marital status, education level, and poverty income ratio, RCI remained an independent risk factor for MI [odds ratio (OR) 95% confidence interval (CI) = 1.68 (1.43–1.96), *P* < .001]. The subgroup analysis indicated that females aged over 60 exhibited a higher risk of MI associated with RCI compared to the overall population. The forward MR analysis included 79 single nucleotide polymorphisms (SNPs) linked to RCI. The results revealed a statistically significant causal relationship: individuals with genetic predisposition to RCI were at a higher risk of developing MI [IVW: OR = 1.09, 95% CI: 1.03–1.14, *P* < .001]. No heterogeneity (*P* > .05) or horizontal pleiotropy (*P* > .05) was detected, and the leave-one-out test did not identify any single SNP that unduly influenced the outcome. Conversely, the reverse MR analysis (which incorporated 78 MI-associated SNPs) did not provide strong evidence for a causal link in the opposite direction [IVW: OR = 0.98, 95% CI: 0.94–1.02, *P* = .39]. These findings suggest a directional causal relationship: RCI may increase the risk of MI, and vice versa, an association that is particularly pronounced in women over 60.

## 1. Introduction

**Figure 1.**
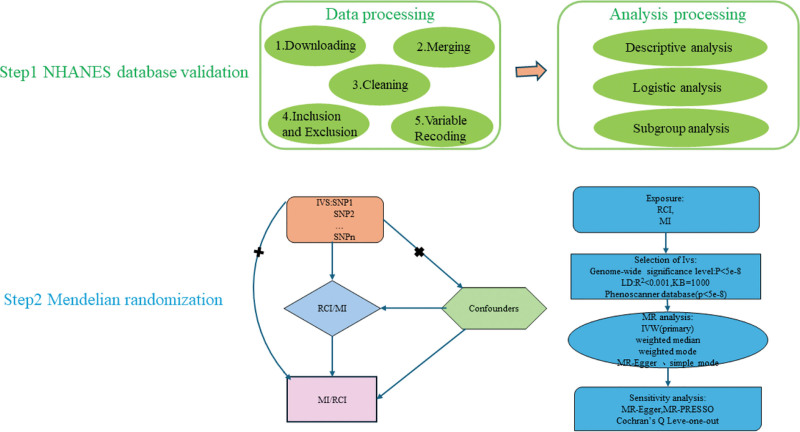
Flowchart of the study workflow.

Rotator cuff injury (RCI) is a common and extremely prevalent disease in clinical practice and is one of the most prominent causes of shoulder pain and activity restriction.^[1]^ The rotator cuff is an important structure composed of the supraspinatus, infraspinatus, subscapularis, teres minor, and their associated tendons, which plays a crucial role in the stability and motor function of the shoulder joint.^[2]^ According to epidemiologic statistics, the current prevalence of RCI is 23% and its incidence increases with age.^[3]^ Myocardial infarction (MI) is a serious cardiovascular event due to acute and persistent ischemia and hypoxia in the coronary arteries, resulting in necrosis of myocardial tissue, which is often manifested clinically as severe and persistent retrosternal pain.^[4]^ MI, often referred to as a heart attack, is a serious cardiovascular event manifested by irreversible damage to myocardial tissue due to blockage of the coronary arteries. Acute MI is one of the leading and most common causes of chronic heart failure globally.^[5]^ MI is one of the diseases with high morbidity and mortality rates globally, placing a significant burden on the healthcare system.^[6]^ Increasing evidence suggests a potential link between musculoskeletal system disorders and cardiovascular events, but the causal link between RCI and MI are unclear.^[7–9]^ To investigate the relationship between RCI and MI, we initially conducted an observational study using data from the US population based on the National Health and Nutrition Examination Survey (NHANES) database. Additionally, we performed a bidirectional 2-sample Mendelian randomization (MR) analysis to discern the causal impact of MI on the risk of RCI from a genetic variation perspective. This study lays a theoretical foundation for the early identification and prevention of individuals with RCI and/or MI. Figure 1 illustrates the study workflow.

## 2. Materials and methods

### 2.1. Epidemiological cross-sectional study

#### 2.1.1. Study design and data sources

**Figure 2.**
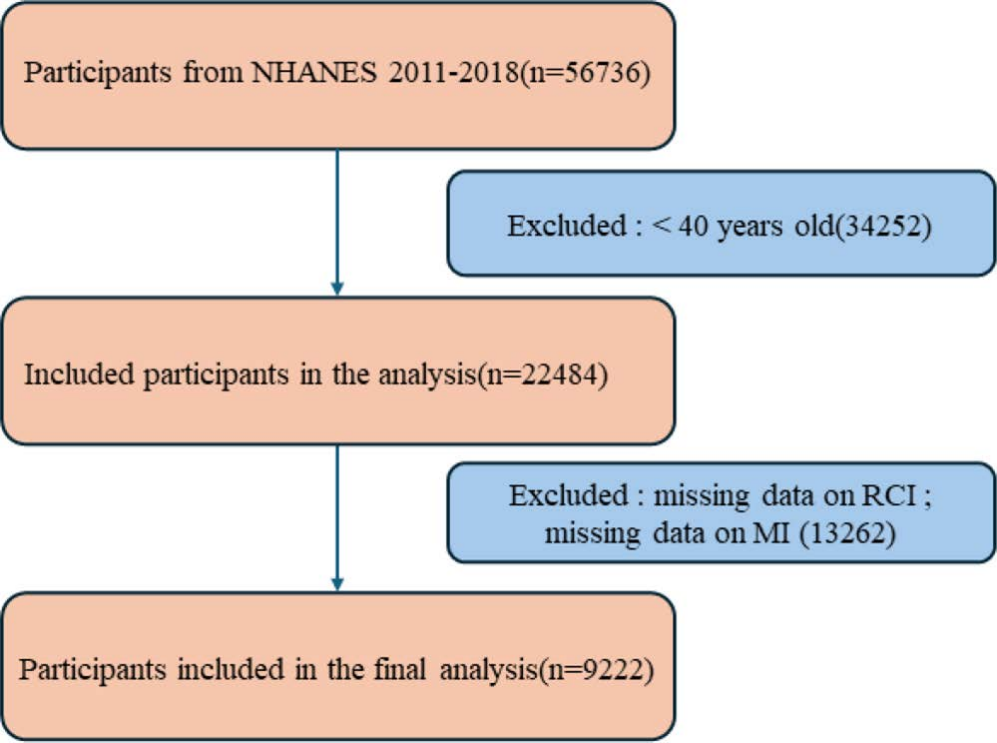
Flowchart of participant selection.

Data for this study were derived from the NHANES, a cross-sectional survey program designed to assess the health and nutritional status of adults and children in the United States.^[10]^ NHANES has received approval from the Institutional Review Board of the National Center for Health Statistics, a division of the Centers for Disease Control and Prevention, which is responsible for generating key national health statistics. Our study integrated data spanning 4 NHANES cycles from 2011 to 2018. In total, 56,736 participants completed demographic interviews, physical examinations, laboratory tests, and health questionnaires. Exclusion criteria were applied as follows: participants younger than 40 years (n = 34,252); and participants with missing data on RCI or MI (n = 13,262). Ultimately, 9222 participants were included in the final analysis. The detailed inclusion and exclusion process is illustrated in Figure 2.

#### 2.1.2. Variables included in the NHANES analysis

The definition of RCI is based on the “Physical Functioning” section of NHANES. Participants who answered “Some difficulty,” “Much difficulty,” or “Unable to do” to either “PFQ061O (Reaching up over head difficulty)” or “PFQ061P (Grasp/holding small objects difficulty)” were defined as suspected RCI cases. The definition of MI is based on the variable MCQ160E in the NHANES “Medical Conditions” questionnaire module. Participants who answered “Yes” to “Ever told you had heart attack” (MCQ160E = 1) were defined as MI patients. Covariates include age, gender, race, poverty income ratio, smoking, education level, and marital status.^[11]^

#### 2.1.3. Statistical analysis

Logistic regression analysis was employed to evaluate the association between RCI and MI.^[12]^ Baseline characteristics were summarized using descriptive statistics, and differences between groups were assessed by chi-square tests or *t* tests as appropriate.^[13]^ MI status served as the dependent variable in a stepwise logistic regression modeling approach adjusting for covariates sequentially. Model 1 was unadjusted; Model 2 adjusted for sex, age, and race; Model 3 further adjusted for education level, marital status, poverty income ratio, and smoking status based on Model 2. Odds ratio (OR) with 95% confidence intervals (CIs) and *P*-values were reported. Additionally, subgroup analyses were conducted to explore the potential modifying effects of sex, age, marital status, smoking, poverty, and education on the association between RCI and MI. All statistical analyses were performed using R software (version 4.5.0; R Foundation for Statistical Computing, Vienna, Austria).

### 2.2. MR analysis

#### 2.2.1. Study design

To explore the potential causal relationship between RCI and MI, we employed a bidirectional 2-sample MR approach. Single nucleotide polymorphisms (SNPs) were selected as instrumental variables (IVs) from publicly available genome-wide association studies (GWAS) datasets. In the forward MR analysis, RCI was treated as the exposure and MI as the outcome, whereas in the reverse MR analysis, MI served as the exposure and RCI as the outcome. To ensure the validity of causal inference and minimize bias, all selected IVs were required to meet the 3 core assumptions of MR: relevance (correlation): the IV must be strongly associated with the exposure of interest (either RCI or MI); independence: the IV should be independent of any known confounding variables that may influence both the exposure and the outcome; and exclusion restriction (exclusivity): the IV must affect the outcome solely through their influence on the exposure, without any alternative biological pathways.^[14]^ By adhering to these assumptions, the MR framework strengthens causal inference, offering a robust tool for disentangling complex relationships between musculoskeletal and cardiovascular conditions.

#### 2.2.2. Data sources

The genetic data used in this MR study were obtained from large-scale, publicly accessible GWAS, each conducted on populations of European ancestry. RCI data were derived from a 2024 Finnish cohort, comprising 390,666 individuals, including 33,117 patients diagnosed with RCI and 357,549 controls. This dataset encompassed approximately 21,325,227 SNPs loci, providing a comprehensive genetic landscape for analysis. For MI, summary-level GWAS data were sourced from a 2021 European study involving a total of 638,000 participants, including 61,000 individuals with MI and 577,000 controls. This dataset contained approximately 8,126,033 SNP loci, offering substantial statistical power for downstream MR analysis. All GWAS data used in this study were obtained from publicly available databases. Ethical approvals had been secured by the original study authors, ensuring that all data usage adhered to institutional and regulatory guidelines for biomedical research involving human subjects. The data sources and information for RCI and MI are shown in Table 1.

**Table 1:** Source of data.

Exposure/outcome	Cases	Control	SNP	Sources
RCI	33,117	357,549	21325227	https://www.finngen.fi/fi
MI	61,000	577,000	8126035	https://www.ebi.ac.uk/gwas/home

MI = myocardial infarction, RCI = rotator cuff injury, SNPs = single nucleotide polymorphisms.

#### 2.2.3. IV selection

Among the genetic variants associated with RCI/MI, when they reached the genome-wide significance level (*P* < 5 × 10^−8^), the variant was selected as an IV. To ensure the independence of selected IV and minimize bias due to linkage disequilibrium (LD), LD pruning was performed using R software, with parameters set at *r*² < 0.001 and an LD window of 10,000 kilobases.^[15]^ To further satisfy the core MR assumptions, especially those of independence and exclusivity, all selected IVs underwent allele harmonization to ensure correct orientation, and were then cross-validated using the PhenoScanner V2 database. During this process, filtering criteria were defined as *P* < 5 × 10⁻⁸ and *r*² > 0.8, thereby strengthening the validity of IVs selection.^[16]^ Additionally, the *F*-statistic for each IV was calculated using the formula:


$$\begin{array}{l} F=\frac{R^{2}(N-2)}{1-R^{2}} \end{array}$$

In this formula, *R*² represents the proportion of RCI/MI variants explained by each IV, while *N* is the GWAS sample size for RCI/MI association. IVs with *F*-statistics > 10 were considered strong instruments, effectively reducing the risk of weak instrument bias and enhancing the robustness of causal inference.^[17]^ Following the harmonization of MI-associated IVs with GWAS summary data for RCI, we further excluded IVs not aligned on the same DNA strand, as well as those with ambiguous alleles or intermediate allele frequencies, in order to prevent strand ambiguity and allele misclassification.^[18]^

### 2.3. Statistical analysis

For MR analysis, the inverse variance weighted (IVW) method was used as the main analytical method.^[16]^ In addition, other complementary methods were used to enhance the robustness of the IVW analysis results, including the MR-Egger method, the weighted median method, the weighted model, and the simple model.^[17]^ When the causal effects obtained from the different methods are consistent, it indicates high confidence in the causal evidence; however, if there is inconsistency in the effect estimates across models, the IVW method has the highest statistical efficacy among the 5 methods.^[18]^ The *P*-value threshold for all MR analyses was <.05 to determine the causal effect of exposure on outcomes. The results of all analyses were expressed as OR and their 95% CI. All MR analyses and tests were performed using the “TwoSampleMR” (version 0.6.8) and “MRPRESSO” (version 1.0) software packages in R software (version 4.5.0).

Given that IVW methods cannot adequately address horizontal pleiotropy and confounders among SNPs and may be affected by outliers or influential SNPs, it is necessary to check for heterogeneity and pleiotropy and to exclude outliers or influential SNPs prior to using IVW methods. We utilized the Cochran *Q* test to check for heterogeneity among genetic variants from different genetic variants, and if *P* < .05, it indicates the presence of heterogeneity.^[19]^ MR-Egger intercept test can be used to detect and assess horizontal pleiotropy, in which the intercept term is a key indicator for determining horizontal pleiotropy, and if the intercept term is greater than zero, it indicates the presence of horizontal pleiotropy.^[20]^ In addition, the MR-PRESSO test identifies and removes outliers and provides corrections after deletion.^[21]^ The leave-one-out method was used to assess the potential impact of individual SNPs on the results of MR analysis.

## 3. Results

### 3.1. Epidemiological cross-sectional study observation and analysis

Participant characteristics were extracted from the NHANES 2011 to 2018 cycles, and a total of 9222 participants were included in the present analysis. Table 2 summarizes the baseline characteristics across the 4 survey cycles. Based on the AWGS criteria, 2680 individuals (29.7%) were classified as having RCI, while 6542 individuals (70.9%) were classified as non-RCI. In the baseline table, significant differences (*P* < .05) were observed between the RCI and non-RCI groups in terms of MI prevalence, age distribution, sex, race, education level, marital status, and smoking behavior. Notably, the prevalence of MI was markedly higher in the RCI group compared to the non-RCI group (11.0% vs 7.2%), suggesting a potential association between RCI and MI. As shown in Table 3, a positive association was observed between RCI and the prevalence of MI. Logistic regression models demonstrated that the ORs for MI in participants with RCI were 1.60 (95% CI: 1.36–1.87), 1.78 (95% CI: 1.52–2.08), and 1.68 (95% CI: 1.43–1.96) in Models 1, 2, and 3, respectively, indicating that RCI may be an independent risk factor for MI.

**Table 2:** Baseline characteristics of participants stratified by the RCI.

Characteristic	Non-RCI (n = 6542)	RCI (n = 2680)	*P*-value
MI, No	6072 (92.8)	2384 (89.0)	<.001
MI, Yes	470 (7.2)	296 (11.0)	
Age 40–60	1546 (23.6)	876 (32.7)	<.001
Age > 60	4996 (76.4)	1804 (67.3)	
Gender, Male	3292 (50.3)	1110 (41.4)	<.001
Gender, Female	3250 (49.7)	1570 (58.6)	
Race, Mexican American	578 (8.8)	282 (10.5)	<.05
Race, Non-Hispanic Black	1604 (24.5)	656 (24.5)	
Race, Non-Hispanic White	2958 (45.2)	1232 (46.0)	
Race, Other	1402 (21.4)	510 (19.0)	
Education, Below high school	1814 (27.7)	908 (33.9)	<.001
Education, High School or above	4728 (72.3)	1772 (66.1)	
PIR, Poor	1260 (19.3)	776 (29.0)	<.001
PIR, Not poor	5282 (80.7)	1904 (71.0)	
Marital, No	2844 (43.5)	1392 (51.9)	<.001
Marital, Yes	3698 (56.5)	1288 (48.1)	
Smoke, No	5474 (83.7)	2094 (78.1)	<.001
Smoke, Yes	1068 (16.3)	586 (21.9)	

PIR = poverty income ratio, RCI = rotator cuff injury.

**Table 3:** Association of RCI with MI.

Model	Non-RCI OR (95% CI)	RCI OR (95% CI)	*P*-value
Model 1	Ref	1.60 (1.38–1.87)	1.4e-09
Model 2	Ref	1.78 (1.52–2.08)	3.72e-13
Model 3	Ref	1.68 (1.43–1.96)	1.13e-10

Model 1: unadjusted; Model 2: adjusted for age, sex and race; Model 3: adjusted for variables included in Model 2 and PIR, smoking, marital, education.

CI = confidence interval, OR = odd ratio, PIR = poverty income ratio, RCI = rotator cuff injury, MI = myocardial infarction.

**Figure 3.**
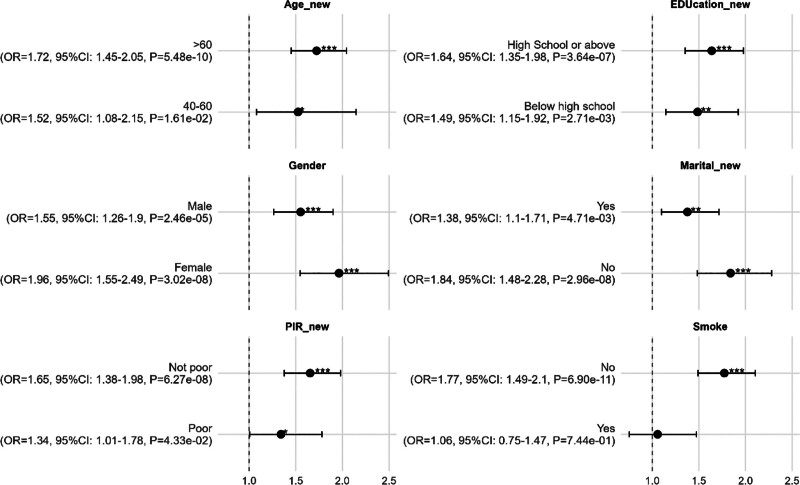
Forest plot of RCI effect on MI (subgroups). * 0.01 ≤ *P* < .05; ** 0.001 ≤ *P* < .01; *** *P* < .001. MI = myocardial infarction, RCI = rotator cuff injury.

To further elucidate the relationship between RCI and MI, subgroup analyses were conducted stratified by age, sex, educational level, poverty status, marital status, and smoking behavior. As shown in Figure 3, the association between RCI and MI remained statistically significant across most subgroups. Notably, a stronger association was observed in participants aged over 60 years (OR = 1.72, 95% CI: 1.45–2.05, *P* = 5.48 × 10⁻¹⁰) and among females (OR = 1.96, 95% CI: 1.55–2.49, *P* = 3.02 × 10⁻⁸). However, in the smoking subgroup, the association did not reach statistical significance (*P* = .744), suggesting that smoking may play a potential modifying role. Collectively, these findings indicate that RCI is more likely to contribute to the development of MI.

### 3.2. The results of bidirectional MR analysis

#### 3.2.1. Influence of RCI on MI

##### 3.2.1.1. Determination of IVs

In this study, we included 79 SNPs that were significantly associated with the risk of RCI. These SNPs did not exhibit LD, as indicated by an *r*² < 0.001, and were excluded from the possibility of being weak IVs, with all *F*-statistics exceeding the threshold of 10. This met the selection criteria previously established for strong IVs. Of these 79 SNPs, 4 were missing when paired with MI data. Importantly, no SNPs were found to be associated with potential confounders or to show horizontal pleiotropy, ensuring the validity of the IVs. The *F*-statistics were assessed, revealing an overall *F*-value of 23.02, with all individual *F*-values exceeding 10, indicating that the selected SNPs had strong predictive power and effectively minimized the risk of confounding. Given the skewness observed in the data, the median was used to describe the central tendency of the *F*-values, ensuring robust estimates.

##### 3.2.1.2. MR analysis

**Figure 4.**
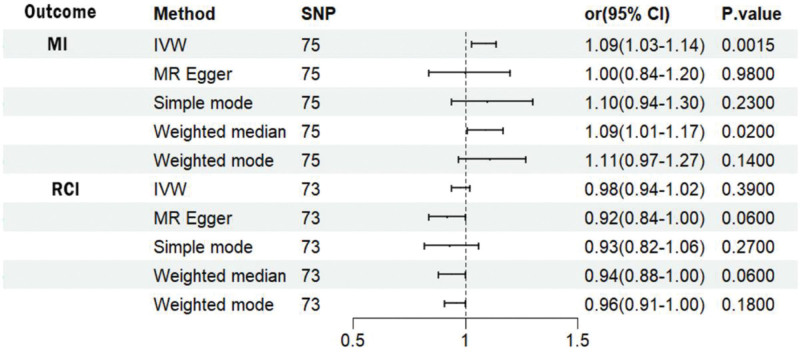
The result of bidirectional MR analysis. MI: the situation of the effect of the exposure factor RCI on the outcome when the outcome is myocardial infarction. RCI: the effect of the exposure factor MI on the outcome when the outcome is rotator cuff injury. MI = myocardial infarction, RCI = rotator cuff injury.

**Figure 5.**
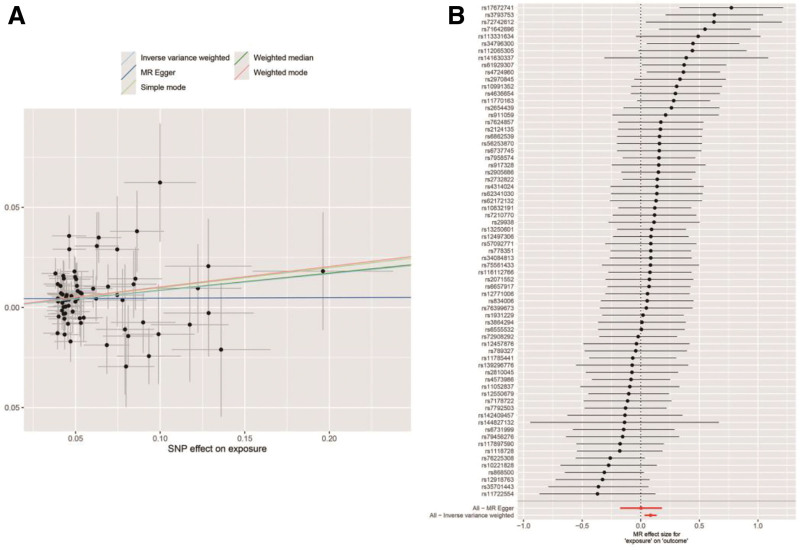
(A) Scatter plot and (B) forest plot.

We performed MR analysis on the selected SNPs, and the results are shown in Figure 4. Based on the IVW method, we found a statistically significant positive causal relationship between RCI risk and MI. The weighted median method also indicated a significant causal effect. However, the MR-Egger method, the simple model, and the weighted model did not show statistically significant causal effects. Weighted and simple models are 2 different approaches to statistical causal effects in MR analysis. The weighted model assigns weights based on the variance of the genetic instruments, while the simple model does not consider variance.^[20]^ Typically, simple models are less accurate, whereas weighted models may also be inaccurate in small samples and are influenced by the degree of association between genetic instruments and exposure and outcome variables.^[21]^ In this study, the lack of statistical significance in the results of the weighted model, the simple model, and the MR-Egger method may be due to the influence of certain genetic tools. Our main approach is based on IVW, which utilizes all valid genetic tools, thus improving statistical power and providing the most accurate and valid results without heterogeneity and horizontal pleiotropy. From the forest plot in Figure 5B we can see that the results of multiple methods are in the same direction, with IVW and median methods being significant, suggesting a positive correlation between RCI and MI. The scatter plot in Figure 5A shows that there are no extreme outliers, and the positive IVW slope indicates a good model fit. Therefore, the results of the weighted model, the simple model, and the MR-Egger method, which differ from the results of the other methods, are considered unreliable and do not significantly affect the overall conclusions of the MR analysis. On the contrary, more weights were assigned to the IVW method, which is widely recognized as the most robust and reliable of the available methods.

##### 3.2.1.3. Sensitivity analysis

**Figure 6.**
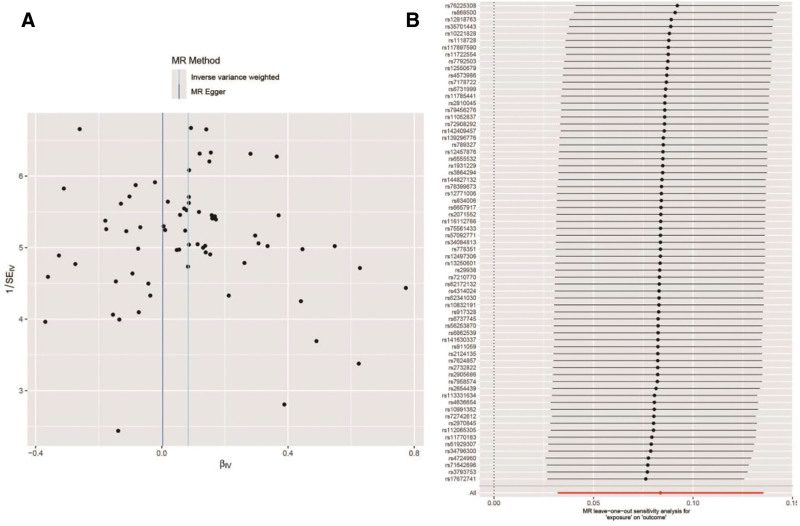
(A) Funnel plot and (B) leave-one-out plot.

We tested all IVs for heterogeneity, using the *Q* statistics to assess the differences between them. The *Q* statistics were calculated to be 87.57 (*P* = .06), indicating no significant heterogeneity. In addition, the MR-PRESSO overall test did not detect any outlier SNP or RCI effects on horizontal pleiotropy in MI, and the MR-Egger intercept test found no evidence of horizontal pleiotropy (Egger intercept = 0.004, *P* = .36). These findings indicate that the study is robust and unlikely to be affected by confounding bias. The funnel plot in Figure 6A shows that the SNP points are evenly distributed on both sides of the IVW regression line, indicating that there is no significant pleiotropy. Sensitivity analysis using the leave-one-out method, which consists of sequentially removing SNPs, recalculating the causal effect of the remaining SNPs, and observing whether the results change with each SNP removal, makes it clear that no single SNP substantially affects the results, demonstrating that the results are reliable (Fig. 6B).

### 3.3. Influence of MI on RCI

#### 3.3.1. Determination of IVs

In this study, we included a total of 78 SNPs significantly associated with the risk of MI. The potential issues of LD and weak IVs were thoroughly addressed during the selection process. Notably, 5 SNPs were excluded from the analysis due to missing data when paired with RCI data. Additionally, no SNPs were found to be associated with any confounders or horizontal pleiotropy, ensuring the robustness of the genetic instruments used. The *F*-statistics for the selected SNPs were examined to ensure the validity of the IVs. The *F*-value was calculated to be 41.04, with all *F*-values exceeding the threshold of 10, which indicates strong IVs and minimal risk of weak instrument bias. Given the skewed distribution of the data, we chose the median to describe the central tendency, ensuring the accuracy of the analysis.

#### 3.3.2. MR analysis

The analysis of the 73 SNPs revealed that the results from the IVW, weighted median, MR-Egger method, simple model, and weighted model methods were highly consistent. There was insufficient evidence to support a causal relationship between MI and the risk of developing RCI.

#### 3.3.3. Sensitivity analysis

**Figure 7.**
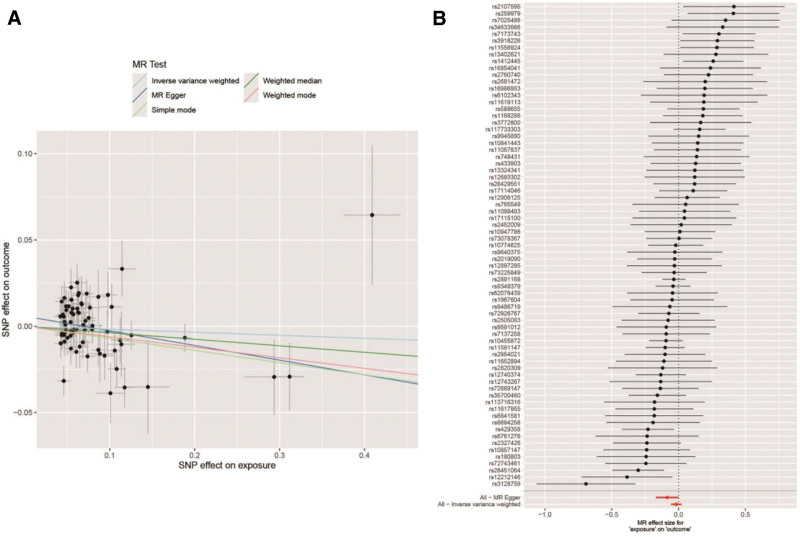
(A) Scatter plot and (B) forest plot.

To assess heterogeneity, we used different methods for the Cochran *Q* test. The results of the IVW method showed a Cochran *Q* value of 0.37 and a *P*-value of .83, while the Cochran *Q* value for the MR-Egger method was 0.24 with a *P*-value of .62, both indicating no significant heterogeneity between the IVs. The forest plot in Figure 7B shows that none of the MR analysis methods found a significant causal relationship between MI and RCI, while each valuation CI crossed the null line. In the Figure 7A scatterplot, the distribution of estimates across IVs was more dispersed with non-significant slopes, further supporting the lack of causality.

## 4. Discussion

This study provides novel and robust evidence supporting a unidirectional causal relationship between RCI and MI. Using both cross-sectional data and bidirectional MR, we demonstrated that individuals with RCI have a significantly increased risk of developing MI. However, the reverse MR analysis did not reveal any causal association from MI to RCI. Additionally, subgroup analysis suggested that the association between RCI and MI is particularly strong in females over the age of 60, indicating a high-risk subgroup worthy of clinical attention.

The causal direction from RCI to MI may be explained by several biological and behavioral mechanisms. First, RCI often results in chronic pain and impaired upper limb function, leading to reduced physical activity levels.^[22]^ Physical inactivity is a well-established risk factor for cardiovascular diseases, contributing to endothelial dysfunction, atherosclerosis progression, and metabolic dysregulation.^[23,24]^ Second, chronic musculoskeletal injuries such as RCI are frequently accompanied by systemic low-grade inflammation. Pro-inflammatory cytokines (IL-6, TNF-α) involved in tendon degeneration can promote vascular inflammation and plaque instability, thereby increasing cardiovascular risk.^[25–27]^ In this context, RCI may serve as both a marker and a contributor to systemic inflammatory burden that predisposes individuals to MI.^[28]^ In contrast, the absence of a causal effect from MI to RCI, as shown in the reverse MR analysis, suggests that MI is unlikely to initiate or contribute to tendon degeneration directly. While MI can result in physical deconditioning and muscle atrophy, it does not typically affect the structural integrity of the rotator cuff.^[29]^ Moreover, the pathology of tendon degeneration is a gradual and localized process influenced by mechanical overload, vascular supply, and age-related matrix changes (factors not directly impacted by cardiac ischemic events). The lack of reverse causality also reinforces the robustness of our forward-direction findings.

Our subgroup analysis revealed that women aged 60 and above exhibited a stronger association between RCI and MI compared to other demographic groups. Several factors may underlie this age- and sex-specific vulnerability. First, postmenopausal women experience a decline in estrogen levels, which has been linked to both tendon degeneration and increased cardiovascular risk.^[30]^ Estrogen plays a protective role in maintaining vascular function and musculoskeletal integrity; its loss may accelerate both tendon wear and atherosclerotic processes.^[31]^ Second, older women often face delays in diagnosis and treatment of both musculoskeletal and cardiovascular conditions, potentially leading to prolonged inflammation and cumulative damage. Additionally, women tend to experience atypical symptoms of MI, which may contribute to under-recognition and undertreatment, further amplifying their risk.^[32]^ Lastly, social and behavioral factors such as lower physical activity levels, higher rates of osteoporosis, and reduced access to rehabilitation services in older women may also contribute to the observed risk amplification.^[33]^ These findings highlight the importance of targeted prevention and integrated care strategies for this vulnerable population.

## 5. Conclusions

This study, through a cross-sectional analysis of the NHANES database and MR, suggests that RCI may increase the risk of MI, indicating a potential causal relationship between the 2. These findings offer new perspectives for early intervention and integrated therapeutic strategies for MI, particularly emphasizing the need for enhanced cardiovascular risk assessment and management in older female patients. These findings require confirmation through additional longitudinal cohort studies utilizing larger sample sizes.

## Acknowledgments

We sincerely thank the GWAS Catalog, the FinnGen consortium, and NHANES for providing open-access datasets that made this study possible. Their efforts in data collection, curation, and sharing have significantly contributed to the advancement of public health and scientific research. We also express our gratitude to the editors and anonymous reviewers of Medicine for their valuable comments and suggestions, which greatly improved the quality of our manuscript.

## Author contributions

**Conceptualization:** Wang Zhi.

**Data curation:** Dang Jianjun.

**Formal analysis:** Dang Jianjun.

**Software:** Xu Liang.

**Visualization:** Yang Xin.

**Writing – original draft:** Wang Zhi.

**Writing – review & editing:** Wang Zhi, Jia Chengming.
